# Rare Occurrence of a Poorly Differentiated Neuroendocrine Tumor of the Bladder

**DOI:** 10.1155/2017/4812453

**Published:** 2017-01-02

**Authors:** Katherine Dowd, Charles Rotenberry, Douglas Russell, Mitchell Wachtel, Werner de Riese

**Affiliations:** ^1^Department of Urology, Texas Tech University Health Sciences Center School of Medicine, Lubbock, TX, USA; ^2^Department of Pathology, Texas Tech University Health Sciences Center School of Medicine, Lubbock, TX, USA

## Abstract

Neuroendocrine tumors rarely occur in the urinary bladder. They can be carcinomatous, subdivided into small cell and large cell pathology. Small cell carcinoma of the bladder is a rarity that may present at an advanced pathologic stage. No treatment regimens have been standardized for local or metastatic disease. Review of the recent literature shows equivalent survival data for localized disease treated with chemoradiotherapy combined with either bladder sparing surgery or radical cystectomy. Patients with significant comorbidities are an additional challenge. We report a case of poorly differentiated neuroendocrine tumor of the bladder, which could not be classified as small or large cell carcinoma, complicated by significant comorbidities. After management with transurethral resection of the tumor, adjuvant chemotherapy, and radiation, the patient is alive and asymptomatic nearly 1 year after initial TURBT with no evidence of disease recurrence.

## 1. Introduction 

Neuroendocrine tumors are neoplasms that rarely occur in the urinary bladder. They may present with asymptomatic hematuria at an advanced pathologic stage and have a poor prognosis. Although they only comprise 0.35–1% of all bladder tumors, they have been extensively studied for gene expression and molecular basis [1, 2]. These tumors can be divided into carcinoid, neuroendocrine carcinoma, or mixed histology. The high-grade neuroendocrine carcinomas can further be subdivided into large cell and small cell classes.

Large cell and small cell tumors have a poor prognosis often reported with fatal outcomes. Large cell carcinoma of the bladder (LCCB) is extremely rare, with less than 25 reported cases as of 2013 [3]. These tumors are encountered so sparingly that no current treatment protocols have been established [4]. Small cell carcinoma of the bladder (SCCB) is poorly differentiated, and more than 95% of cases present at an advanced local stage [5]. SCCB has had a significant rise in incidence from 0.05 to 0.14 cases per 100000 individuals from 1991 to 2005 in the United States, perhaps due to the aging population [1]. Patients are often of Caucasian descent, in their seventh or eighth decade of life, and have a history of smoking [1]. In contrast to their pulmonary counterpart, SCCB is rarely associated with neoplastic syndromes [6]. These neuroendocrine tumors are chemotherapy sensitive and are often managed with a multidisciplinary approach due to their highly malignant potential. In this report, we present a case of a poorly differentiated neuroendocrine carcinoma of the bladder.

## 2. Case Presentation

**Figure 1:**
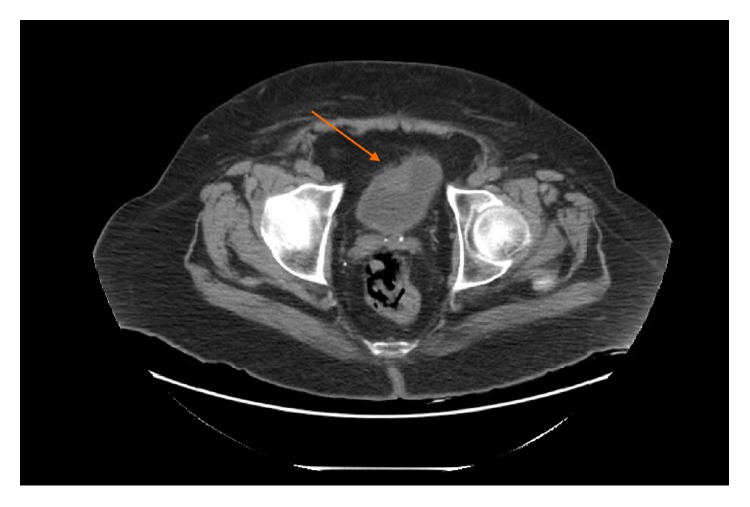
3.2 × 1.7 × 2.7 cm mass in the anterior wall of the urinary bladder.

A 58-year-old Caucasian male with a past medical history of hypertension, type II diabetes mellitus, chronic kidney disease stage IV on peritoneal dialysis, stroke, and congestive heart failure presented to an outside clinic with asymptomatic hematuria. CT scan showed a 3.2 × 1.7 × 2.7 cm mass in the anterior wall of the urinary bladder at that time (Figure 1).

**Figure 2:**
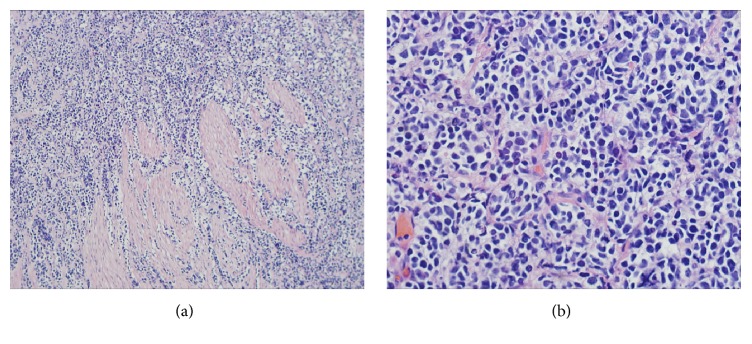
(a) Dark tumor cells fill stroma, splaying pink smooth muscle bands to the left (H&E, 100x). (b) Tumor nuclei are irregular, with scant cytoplasm, with some showing spots of nuclear clearing suggestive of salt and pepper, with some tumor cells juxtaposed to suggest molding, and with many pyknotic nuclei, indicative of a rapidly growing tumor.

He underwent transurethral resection of the bladder tumor (TURBT). Pathology revealed poorly differentiated neuroendocrine tumor with invasion into the muscularis propria and no lymphovascular invasion. Tissue staining showed cells almost entirely lacking definable cytoplasm, with variably sized nuclei, aberrant mitosis, and invasion into the muscle. Dark tumor cells filled the tissue stroma, splaying pink smooth muscle bands (H&E, 100x) (Figure 2(a)). Tumor nuclei were irregular with scant cytoplasm, showing spots of nuclear clearing suggestive of “salt and pepper” chromatin. Some cells were juxtaposed to suggest molding, with many cells containing pyknotic nuclei, indicative of a rapidly growing tumor (Figure 2(b)).

Immunohistochemical staining showed synaptophysin positivity and was Ki-67 positive in >90% of the cells. All other immunohistochemical stains were negative. Pathologists debated the histological findings, and it was determined that the findings of negative vimentin and CD45 and positive synaptophysin and Ki-67 were suggestive of poorly differentiated neuroendocrine carcinoma. He was referred to our urology service to coordinate care with discussion of options for chemotherapy, radiation, and surgery.

Due to his significant comorbidities, he was counseled to undergo systemic chemotherapy with radiation without further surgical intervention. MRI and fluorodeoxyglucose PET/CT scans after TURBT were negative for metastatic disease despite muscular invasion and positive margins of the primary resection. Final staging was determined as pT2b, N0, M0. Patient completed 33 fractions of radiation to the pelvis and bladder utilizing intensity-modulated radiotherapy technique for a total dose of 5940 centigray combined with four cycles of carboplatin/etoposide. He underwent in-clinic cystoscopy six months after TURBT, which showed the previous resection site at posterior bladder wall near the dome with necrotic-appearing tissue but no active tumor. The patient was then scheduled for repeat TURBT with random bladder biopsies. Pathology returned as benign for all biopsies.

## 3. Discussion 

Neuroendocrine tumors of the bladder are often first diagnosed in an advanced stage and have a poor prognosis. A 2013 review of the Surveillance, Epidemiology, and End Results Database (SEER) revealed 663 patients with SCCB and median overall survival of only 12 months [7]. Patients with SCCB rarely survive past two years, and patients with LCC fare even worse. Currently, there is no consensus on stage-related treatment regimens; however, the National Cancer Care Network set recommendations for SCCB, including neoadjuvant or adjuvant chemotherapy combined with a form of local treatment such as radical cystectomy or radiation [8]. The first SCCB was described in 1981, and the first LCC was reported in 1986 [3]. Historically, these tumors were deemed “high grade” and treated with cystectomy with poor results [3]. The first long-term outcomes were seen in 1999, when SCCB was treated similarly to that of its lung counterpart. A reported 44% survival rate was seen at 5 years when adding multiagent chemotherapy and radiation [9].

LCCB is rare but therapeutic success has been seen in select cases of localized disease treated with neoadjuvant or adjuvant chemotherapy plus aggressive surgery [3]. Chemotherapy is currently used for metastatic disease [3, 10]. Most SCCB cases present with asymptomatic hematuria (88%) and a smoking history (65%) [11]. Previously, reports suggested that adjuvant chemotherapy plus radical cystectomy produced superior survival rates [12]. A study of 625 patients with locoregional neuroendocrine tumor in the National Cancer Database in 2014 demonstrated a more favorable outcome for patients treated with neoadjuvant chemotherapy followed by radical cystectomy; a three-year survival rate of 53% was reported in this group versus 23% for bladder sparing therapy without chemotherapy [13].

Because most treatments for SCCB have not been proven through clinical research, the first phase II clinical trial for surgically resectable SCCB is groundbreaking. By adding a neoadjuvant chemotherapy regimen, groups of patients experienced longer disease control and overall survival, especially with patients who had muscle invasion. The chemotherapy regimen consisted of alternating doublet therapy with ifosfamide plus doxorubicin and etoposide plus cisplatin. Patients with cT2N0M0 experienced an 80% 5-year survival rate with their 4-cycle chemotherapy regimen plus radical cystectomy and lymph node dissection. This trial also highlighted the importance of brain imaging, as up to 50% of patients with bulky tumors experienced brain metastasis. Because of the high rate of recurrence that was seen in bladder sparing surgery, this trial recommended cystectomy. Overall, this study emphasizes the importance of adding a chemotherapy regimen, before surgery if possible, with cystectomy as the main mode of tumor resection for good surgical candidates [14].

More recently, a multicenter Rare Cancer Network study of 107 patients uncovered that, in nonmetastatic SCCB, transurethral resection alone had no statistically significant difference in outcomes compared to radical cystectomy, as long as both groups received systemic chemotherapy [15]. Therefore, conservative therapy with TURBT plus chemotherapy may be a reasonable option for patients with poor performance status.

Surveillance for neuroendocrine tumors remains similar to surveillance for urothelial cell carcinoma (UCC) because no follow-up protocol currently exists. Vigilant surveillance with cystoscopy is important, usually done every three to six months after bladder sparing surgery due to the high risk of reoccurrence; this risk may be attributable to the UCC component of these tumors [16]. Follow-up imaging is also imperative, as microscopic metastasis is common. Imaging should be repeated within the first year, with inclusion of both the upper and the lower tract as these patients are at high risk [16]. Brain imaging as well as neurologic exam should be considered due to the high frequency of brain metastasis, especially in bulky tumors [14]. Brain metastasis can occur in as high as 40% of cases, reducing life expectancy to less than two months [8]. Repeat brain imaging with MRI may be necessary to evaluate new metastatic growth.

A clinical database for neuroendocrine tumors of the urinary bladder and prospective studies to determine survival patterns based on treatment regimens need to be established in order to verify the best treatment regimen for this rare but devastating malignancy.

## 4. Conclusion

Poor surgical candidates with small cell carcinomas of the bladder may benefit from bladder sparing surgery with adjuvant chemotherapy and radiation. In our case, management with TURBT was followed by radiation and platinum-based chemotherapy due to significant comorbidities. This patient is alive and asymptomatic nearly 1 year after initial TURBT with no evidence of disease recurrence.
